# Diabetic foot ulcers risk prediction in patients with type 2 diabetes using classifier based on associations rule mining

**DOI:** 10.1038/s41598-023-47576-w

**Published:** 2024-01-05

**Authors:** Nasrin Piran, Maryam Farhadian, Ali Reza Soltanian, Shiva Borzouei

**Affiliations:** 1 Department of Biostatistics, School of Public Health, Hamadan University of Medical Sciences, Hamadan, Iran; 2 Department of Biostatistics, Research Center for Health Sciences, School of Public Health, Hamadan University of Medical Sciences, Hamadan, Iran; 3 Department of Biostatistics, Modeling of Noncommunicable Diseases Research Center, School of Public Health, Hamadan University of Medical Sciences, Hamadan, Iran; 4 Department of Endocrinology, Hamadan University of Medical Science, Hamadan, Iran

**Keywords:** Diseases, Endocrinology, Risk factors

## Abstract

Identifying diabetic patients at risk of developing foot ulcers, as one of the most significant complications of diabetes, is a crucial healthcare concern. This study aimed to develop an associative classification model (CBA) using the Apriori algorithm to predict diabetic foot ulcers (DFU). This retrospective cohort study included 666 patients with type 2 diabetes referred to Shahid Beheshti Hospital in Iran between April 2020 and August 2022, of which 279 (42%) had DFU. Data on 29 specific baseline features were collected, which were preprocessed by discretizing numerical variables based on medical cutoffs. The target variable was the occurrence of DFU, and the minimum support, confidence, and lift thresholds were set to 0.01, 0.7, and 1, respectively. After data preparation and cleaning, a CBA model was created using the Apriori algorithm, with 80% of the data used as a training set and 20% as a testing set. The accuracy and AUC (area under the roc curve) measure were used to evaluate the performance of the model. The CBA model discovered a total of 146 rules for two patient groups. Several factors, such as longer duration of diabetes over 10 years, insulin therapy, male sex, older age, smoking, addiction to other drugs, family history of diabetes, higher body mass index, physical inactivity, and diabetes complications such as proliferative and non-proliferative retinopathy and nephropathy, were identified as major risk factors contributing to the development of DFU. The CBA model achieved an overall accuracy of 96%. Also, the AUC value was 0.962 (95%CI 0.924, 1.000). The developed model has a high accuracy in predicting the risk of DFU in patients with type 2 diabetes. The creation of accurate predictive models for DFU has the potential to significantly reduce the burden of managing recurring ulcers and the need for amputation, which are significant health concerns associated with diabetes.

## Introduction

The increasing prevalence of diabetes is a considerable global health concern. According to the World Health Organization (WHO) Type 2 diabetes rates have raised worldwide across all income levels. Diabetic foot (DF) is a severe complication for diabetes patients, with a global prevalence of 6.3%, often resulting in a high amputation rate^1–3^. Individuals with diabetes have a 25% chance of developing DF during their lifetime. The mortality rate associated with DF development is approximately 5% within the first 12 months and 42% within five years^4,5^. The annual incidence of diabetic foot ulcers (DFUs) worldwide ranges from 1.9 to 26.1 million. The prevalence of DFUs varies significantly between countries and regions, spanning 1.5% to 16.6%^6,7^.

DFUs can have serious consequences, including a high rate of disability, mortality, and recurrence, as well as high treatment costs and prolonged hospitalization^8–12^. Poor prognosis imposes a significant financial burden on patients, their families, and medical and health systems. To prevent these complications, diabetic patients with a “high-risk” foot must regularly see a doctor, take costly medications, and take personal responsibility for their health^13,14^.

Delaying specialist evaluation for DFUs can result in more severe ulcers, lower cure rates, and more hospitalizations. Clinical guidelines recommend annual foot screening of all diabetic patients to identify those at high risk for developing foot ulcers and prevent amputations^15,16^. High-risk individuals can be identified through a clinical examination of the feet. Identifying individuals likely to develop ulcers allows for targeted preventive treatments. Early screening and prediction of DFUs in high-risk groups is a crucial step in managing the prognosis of diabetic patients. Accurate prediction of diabetic foot ulcer risk can significantly reduce the burden of chronic wounds and amputations^17,18^.

Machine learning is a subfield of artificial intelligence that enables systems to automatically learn patterns from data and enhance clinical decision-making. Utilizing machine learning techniques, data-driven medical decision-making systems can provide valuable and insightful information in clinical and diagnostic areas. A well-designed predictive model can assist medical professionals and patients in preventative care strategies^19^.

Effective medical diagnosis relies on knowledge discovery from medical databases. Therefore, data mining is a more suitable approach for medical studies. Data mining involves extracting information from databases and creating clear and understandable descriptions of patterns. Among unsupervised data mining methods, association rule mining is one of the most popular and effective techniques for extracting useful information and discovering relationships between elements in large amounts of data stored in databases^20,21^. Association rule mining has been used in various medical applications, such as identifying patterns in disease progression. Association rules specify conditions that frequently occur together in a given dataset. The extracted rules describe the presence of certain features based on other features^22,23^. The Apriori algorithm is a powerful tool for exploring frequent itemsets to discover association rules, which are then used as the basis for other discovery algorithms. The algorithm derives its name from the fact that it uses prior knowledge about the properties of frequent itemsets. The processing involves identifying significant rules among frequent patterns, which are extracted by setting support and confidence thresholds^24^. The association rules using the Apriori algorithm produces interpretable and intuitive results, providing information about general trends in the database.

Given the limited number of studies on extracting knowledge from data related to DFUs using association rules, this study aimed to develop an association classification model utilizing the Apriori algorithm. The model will predict the risk of DFUs based on the collection of demographic, clinical, and laboratory variables. Also,

## Material and methods

### Material

This study was a retrospective cohort analysis involving all patients consecutively referred to Shahid Beheshti Hospital (Hamadan Province, Iran) from April 2020 to August 2022. Data on 29 specific features were collected through a checklist from 666 patients with type 2 diabetes, of which 279 (42%) patients had diabetic foot ulcers (DFUs).

All patients previously diagnosed with diabetes (type 2) registered between April 2020 and August 2022 was included in the study. Inclusion criteria were as follows: age > = 25 years; meeting the ADA (American Diabetes Association) guidelines 2023 for diagnostic criteria for diabetes; haemoglobin A1c (HbA1c) ≥ 6.5% at any time before first hospitalisation; fasting plasma glucose (FPG) ≥ 126 mg/dL or 2h post-challenge plasma glucose (2h PCPG) ≥ 200 mg/dL; patient with classic symptoms of hyperglycemia or hyperglycemic crisis, a random plasma glucose $200 mg/dL (11.1 mmol/L), taking antidiabetic medication^25^.

International Working Group on the Diabetic Foot (IWGDF) guidelines were used to identify patients hospitalized with DFU. A foot ulcer was defined as a full-thickness lesion below the ankle, irrespective of the presence of neuropathy and/or peripheral arterial disease. DFUs were defined as wounds, infections or destruction of deep tissues in the lower limb below the ankle^26–28^. Individuals with more than one ulcer at baseline were also included as DFUs. The Wagner classification system was used to evaluate the severity of DFUs^29^. All patients with type 2 diabetes mellitus who were on treatment and had at least one episode of foot ulceration during their treatment, with at least Wagner stage 1 or above, were considered 'DFUs or claas1'. The diagnosis of the disease is made using the classification done at the time of the patient's initial admission to the hospital.

The present study was designed as a classification task because we developed a predictive associative CBA model to identification of diabetic patients at risk of DFU. The binary target variable or consequent in the present study is the occurrence of DFUs in patients (class 1: patients with type 2 diabetes with DFUs, and class 0: patients with type 2 diabetes without DFUs).

The logistic regression model was also used as a conventional classical competitor to compare the predictive performance of CBA model. It should be noted that the independent or input variables and the dependent (or target) variable in the logistic regression model were similar to those in the CBA model. A significance level of 0.05 was used. It should be noted that the CBA algorithm and the logistic regression model used the same training and test parts in order to obtain comparable results.

Authors confirm that all experiments were performed in accordance with relevant guidelines and regulations. The research ethics committee of Hamadan University of Medical Sciences approved the study (IR.UMSHA.REC.1401.014), and informed consent was waived due to the retrospective design and use of anonymized clinical data.

### Methods

#### Association rule mining

Association rule mining as a data mining method utilized to identify concealed associations, frequent patterns, and correlations within data. This technique is based on the concept of if–then statements, which establish a relationship between two variables, e.g., if A occurs, then B occurs. The antecedent in this statement represents the if-part, and the consequent represents the then-part.

Two measures, support and confidence, are utilized to assess statistical significance and strength of a rule, respectively. Support (AUB) represents the proportion of records in the dataset that contain both A and B (A → B). Confidence calculated by determining the percentage of records in the dataset that includes A and B. In addition, lift, another criterion, is typically used to compare expected and actual confidence. Lift measures how often the if–then statement was anticipated to be true. If the lift value is greater than 1, it suggests that the rule body and rule head co-occur more frequently than would be expected by chance. This implies that the occurrence of the rule body exerts a positive influence on the occurrence of the rule head^30^.

$$ {\text{Support }}\left( {\text{I}} \right) = \left( {\text{Number of transactions containing item I}} \right)/\left( {\text{Total number of transactions}} \right) $$

$$ {\text{Confidence}} = \left( {\text{Number of transactions containing I1 and I2}} \right)/\left( {\text{Number of transactions containing I1}} \right) $$

$$ {\text{Lift}}\left( {{\text{I1}} \to {\text{I2}}} \right) = ({\text{Confidence}}\left( {{\text{ I1}} \to {\text{I2}}} \right)/\left( {{\text{Support}}\left( {{\text{I2}}} \right)} \right) $$

#### Apriori algorithm

The Apriori algorithm is a popular method for mining association rules from transaction data. It involves identifying frequent itemsets as a basis for creating association rules. A frequent itemset refers to a set of items that meets a minimum threshold of support and confidence. Typically, association rules are considered interesting if they satisfy both a minimum support and a minimum confidence threshold, which can be set by users or domain experts^31^. The Apriori algorithm can be broken down into the following steps:

- Step 1: Set a minimum threshold for support and confidence.
- Step 2: Identify all subsets of transactions that meet the minimum support threshold.
- Step 3: Generate all rules for the frequent itemsets that meet the minimum confidence threshold.
- Step 4: Sort the rules by decreasing lift.

#### Classification based on associations (CBA)

Associative classification is a type of association rule mining that focuses only on the class attributes on the right side of the rule (consequence). CBA (Classification Based on Associations) is a method that utilizes association rule techniques to classify data, and it has shown to be more accurate than traditional classification techniques. However, CBA is sensitive to the minimum support threshold, as setting a lower threshold can lead to a large number of rules being generated. To address this, Liu et al. proposed a CBA method that uses an Apriori approach to generate classification rules. Their method has been modified by others and has been shown to be effective in generating accurate classification rules^32,33^.

Association rule learning is typically applied to categorical data. Therefore, for numerical variables, data discretization was performed. This involved converting numerical attributes into categorical variables using established medical cutoffs. After the data preparation and cleaning phase, association rules were generated from the processed data. The Apriori algorithm was used to extract the supervised rules, and then rules with the highest confidence level and expected accuracy were selected. The “arules” and “arulesViz” package in the R software was used for extracting the rule mining.

The rule base for the Apriori algorithm is generated using the entire sample present in the databases. However, to create a diagnostic classification model using the CBA algorithm, it is necessary to use separate sets of training and testing data. Accordingly, 80% of the data was allocated to the training set, and the remaining 20% was used for testing and not used for optimizing the model during development. The performance of the associative classification model was evaluated using the accuracy measure. The binary target variable or consequent in the present study is the occurrence of DFUs in patients (class 1: patients with type 2 diabetes with DFUs, and class 0: patients with type 2 diabetes without DFUs). In this analysis, the minimum support threshold was set to 0.01, and the minimum confidence threshold was set to 0.7. Additionally, the minimum lift threshold was set to 1.

## Results

Table 1 displays the frequency distribution of the investigated variables in diabetic patients stratified by the presence or absence of DFUs. Continuous variables are presented as mean ± standard deviation, while categorical variables reported as the number and percentage.

**Table 1:** Baseline characteristics of the study patients, categorized by whether or not developed a foot ulcer.

Variables	The studied groups (N = 666) (mean ± SD)		*P* value*
	DFUs	No-DFUs	
Gender, n (%)			< 0.001
Male	185 (66.3%)	182 (47.0%)	
Female	94 (33.7%)	205 (53.0%)	
Age (mean ± SD)	63.34 ± 13.22	61.34 ± 12.27	0.046
Age group, n (%)			0.175
Middle aged (25–45 years)	33 (11.8%)	38 (9.8%)	
Senior( 46–65 year	142 (50.9%)	176 (45.5%)	
Old (> 66 years)	104 (37.3%)	173 (44.7%)	
Marital Status, n (%)			0.129
Single	6 (2.2%	3 (0.8%)	
Married	273 (97.8%)	384 (99.2%)	
Education, n (%)			0.135
Illiterate	192 (68.8%)	266 (68.7%)	
Diploma	82 (29.4%)	101 (26.1%)	
Bachelor’s degree	3 (1.1%)	12 (3.1%)	
Master’s degree and higher	2 (0.7%)	8 (2.1%)	
Employment Status, n (%)			0.813
Unemployed	209 (74.9%)	293 (75.7%)	
Employed	70 (25.1%)	94 (24.3%)	
BMI (mean ± SD)	26.97 ± 4.63	26.14 ± 5.44	0.039
BMI group, n (%)			0.099
Under weight (< 18.5)	8 (2.9%)	25 (6.5%)	
Normal weight (18.5–24.9)	89 (31.9%)	134 (34.6%)	
Over weight (25–29.9)	112 (40.1%)	149 (38.5%)	
Obese (> = 30)	70 (25.1%)	79 (20.4%)	
Diabetic treatment, n (%)			< 0.001
Drug	123 (44.1%)	250 (64.6%)	
Injection	156 (55.9%)	137 (35.4%)	
Duration of diabetes, n (%)			< 0.001
Less than 10 years	122 (43.7%)	273 (71.3%)	
More than 10 years	157 (56.3%)	110 (28.7%)	
History of other diseases, n (%)			0.200
Kidney	42 (15.1%)	39 (10.1%)	
Heart	52 (18.6%)	86 (22.2%)	
Kidney & heart	45 (16.1%)	58 (15.0%)	
No	140 (50.2%)	204 (52.7%)	
Smoking, n (%)			< 0.001
No	231 (82.8%)	361 (93.3%)	
Yes	48 (17.2%)	26 (6.7%)	
Addiction to other drugs, n (%)			< 0.001
No	222 (79.6%)	361 (93.3%)	
Yes	57 (20.4%)	26 (6.7%)	
Physical activity, n (%)			0.011
No	264 (94.6%)	380 (98.2%)	
Yes	15 (5.4%)	7 (1.8%)	
Family history of diabetes, n (%)			< 0.001
No	221 (79.2%)	370 (95.6%)	
Yes	58 (20.8%)	17 (4.4%)	
Family history of DFU, n (%)			0.001
No	271 (97.1%)	387 (100%)	
Yes	8 (2.9%)	0 (0.0%)	
Visiting the doctor regularly, n (%)			< 0.001
No	91 (32.6%)	195 (50.5%)	
Yes	187(67.4%)	192(49.5%)	
LDL (mean ± SD)	64.94 ± 32.01	82.22 ± 31.71	< 0.001
LDL group, n (%)			0.002
Optimal (< 100 mg/dl)	245 (87.8%)	301 (77.7%)	
Near optimal (100–129 mg/dl)	16 (5.7%)	58 (15.0%)	
Borderline high (130–159 mg/dl)	10 (3.6%)	18 (4.7%)	
High (160–189 mg/dl)	8 (2.9%)	10 (2.6%)	
HDL (mean ± SD)	52.61 ± 17.34	38.59 ± 13.56	< 0.001
HDL group, n (%)			< 0.001
Low (< 40 mg/dl)	89 (31.9%)	256 (66.1%)	
Borderline high (40–60 mg/dl)	96 (34.4%)	85 (22.0%)	
High (> 60 mg/dl)	94 (33.7%)	46 (11.9%)	
TC (mean ± SD)	126.12 ± 40.98	116.64 ± 28.78	0.001
TC group, n (%)			0.003
Acceptable (< 200 mg/dl)	257 (92.1%)	378 (97.7%)	
Borderline high (200–239 mg/dl)	14 (5.0%)	7 (1.8%)	
High (> 240 mg/dl)	8 (2.9%)	2 (0.5%)	
TG (mean ± SD)	124.50 ± 49.35	112.80 ± 51.62	0.003
TG group, n (%)			0.025
Normal (< 150 mg/dl)	255 (91.4%)	341 (88.1%)	
Borderline high (150–199 mg/dl)	10 (3.6%)	7 (1.8%)	
High (200–499 mg/dl)	14 (5.0%)	39 (10.1%)	
Diastolic BP (mean ± SD)	77.34 ± 11.11	78.43 ± 11.31	0.217
Diastolic BPgroup, n (%)			0.064
Normal (< 80 mmHg)	201(72.8%)	297(76.7%)	
Pre hypertension (80–89 mmHg)	29(10.5%)	49(12.7%)	
Hypertension(> = 90 mmHg)	46(16.7%)	41(10.6%)	
Systolic BP (mean ± SD)	126.91 ± 21.42	124.67 ± 18.68	0.152
Systolic BP group, n (%)			0.156
Normal (< 120 mmHg)	130(47.1%)	210(54.3%)	
Pre hypertension (120–139 mmHg)	84(30.4%)	108(27.9%)	
Hypertension (> = 140 mmHg)	62(22.5%)	69(17.8%)	
Fasting blood sugar (mean ± SD)	234.59 ± 201.30	254.38 ± 80.86	0.081
Fasting blood sugar group, n (%)			0.160
Normal (60–99 mg/dl)	6 (2.1%)	5 (1.3%)	
Pre-diabetes (100–125 mg/dl)	18 (6.5%)	14 (3.6%)	
Diabetes (> 126 mg/dl)	255 (91.4%)	368 (95.1%)	
Blood sugar after breakfast (mean ± SD)	280.00 ± 90.72	288.74 ± 95.02	0.233
Blood sugar after breakfast group, n (%)			0.150
Normal (< 140 mg/dl)	15 (5.4%)	12 (3.1%)	
Pre-diabetes (140–199 mg/dl)	27 (9.7%)	51 (13.2%)	
Diabetes (> 200 mg/dl)	237 (84.9%)	324 (83.7%)	
Proliferative retinopathy, n (%)			0.038
No	224 (80.3%)	334 (86.3%)	
Yes	55 (19.7%)	53 (13.7%)	
Non proliferative retinopathy, n (%)			< 0.001
No	95 (34.1%)	190 (49.1%)	
Yes	184 (65.9%)	197 (50.9%)	
Diabetic nephropathy, n (%)			0.119
No	200 (71.7%)	298 (77.0%)	
Yes	79 (28.3%)	89 (23.0%)	
Diabetic neuropathy, n (%)			< 0.001
No	253 (90.7%)	375 (97.2%)	
Yes	26 (9.3%)	11 (2.8%)	
Cardiovascular events, n (%)			0.713
No	180 (64.5%)	255 (65.9%)	
Yes	99 (35.5%)	132 (34.1%)	
Cerebrovascular events, n (%)			0.113
No	274 (98.2%)	383 (99.5%)	
Yes	5 (1.8%)	2 (0.5%)	

*Chi Square Test.

*DFUs* diabetic foot ulcer.

The results based on univariate analysis using Chi-square test indicate a significant statistical relationship between gender and the occurrence of DFU. Among the individuals with DFUs, 185 patients (66.3%) were male, while in diabetic patients who had not yet developed DFUs, 182 patients (47%) were male.

There is a significant relationship between age and the occurrence of DFU, with the mean and standard deviation of age for patients with DFUs being 63.34 ± 13.22 years, and for those without foot ulcers being 61.34 ± 12.27 years. Additionally, body mass index (BMI) is associated with the occurrence of DFUs, with the mean and standard deviation of BMI for patients with DFUs being 26.97 ± 4.63 kg/m^2^, and for those without DFUs being 26.14 ± 5.44 kg/m^2^.

There is a significant association between the type of diabetes treatment and the occurrence of DFU (*p* < 0.05). Among the patients with DFUs, 123 patients (44.1%) were using anti-diabetic pills and 156 patients (55.9%) were using insulin therapy.

The duration of diabetes is significantly associated with the risk of developing DFUs (*p* < 0.05). Among the patients with DFUs, 157 patients (56.3%) had a history of diabetes for more than 10 years, while among the diabetic patients who had not yet developed DFUs, 110 patients (28.7%) had a history of diabetes for more than 10 years.

Although there was no significant statistical association between comorbidities that a diabetic individual may have (kidney disease, heart disease, kidney and heart disease, and other diseases) and the occurrence of DFUs (*p* > 0.05), out of the 279 individuals with foot ulcers, 42 patients (15.1%) had kidney disease, 52 patients (18.6%) had heart disease, 45 patients (16.1%) had both kidney and heart disease, and finally, 140 patients (50.2%) had other diseases.

There is a significant association between smoking, drug addiction, physical activity, and the occurrence of DFUs (*p* < 0.05). Among the individuals with DFUs, 48 patients (17.2%) were smokers and 57 patients (20.4%) were addicted to other drugs, and 222 patients (79.6%). Also, 264 patients (94.6%) did not engage in physical activity.

Family history of diabetes, family history of DFUs, and regular visits to a doctor, proliferative retinopathy, non-proliferative retinopathy, diabetic neuropathy, LDL, HDL cholesterol, total blood cholesterol, triglycerides, have a significant association with DFUs (*p* < 0.05). However, there is no significant association between systolic and diastolic blood pressure, fasting blood glucose levels, 2-h postprandial glucose levels, diabetic nephropathy, cardiovascular events, and cerebrovascular events with the occurrence of DFUs (*p* > 0.05).

By implementing the CBA algorithm on the data, 146 rules with the minimum degree of support value and the minimum degree of confidence value equal to 1% and 70%, respectively, were identified separately for two groups of patients with and without diabetic foot ulcers. These rules are presented in the Tables 2 and 3.

**Table 2:** Rule generated by the CBA algorithm for patients belongs to DFUs class.

#	Rules	Support	Confidence	Lift
1	Age = 46–65 Years, Duration of diabetes = > 10 Years, Diabetic treatment = Injection, Addiction to other drugs = Yes ⟹ DFU	0.02	1.00	2.33
2	Gender = Male, Diabetic treatment = Injection, Visiting the doctor regularly = No, Family history of diabetes = Yes ⟹ DFU	0.04	1.00	2.33
3	Duration of diabetes ⟹ 10 Years, HDL = High, Bs2hpp = Diabetes, Non proliferative retinopathy = Yes ⟹ DFU	0.04	1.00	2.33
4	Duration of diabetes = > 10 Years, Visiting the doctor regularly = Yes, Smoking = Yes, Bs2hpp = Diabetes ⟹ DFU	0.03	1.00	2.33
5	Gender = Male, Duration of diabetes = > 10 Years, Diabetic treatment = Injection, HDL = High ⟹ DFU	0.03	1.00	2.42
6	Gender = Male, Duration of diabetes = > 10 Years, Diabetic treatment = Injection, HDL = High ⟹ DFU	0.02	1.00	2.33
7	Duration of diabetes = > 10 Years, Diabetic treatment = Injection, Family history of diabetes = Yes, Bs2hpp = Diabetes ⟹ DFU	0.02	1.00	2.33
8	Addiction to other drugs = Yes, HDL = High, TG = Normal, Non proliferative retinopathy = Yes ⟹ DFU	0.02	1.00	2.33
9	Duration of diabetes = > 10 Years, Bs2hpp = Diabetes, Proliferative retinopathy = Yes, Non proliferative retinopathy = Yes ⟹ DFU	0.02	1.00	2.33
10	Age = 46–65 Years, BMI = Obese, TG = Normal, Diabetic nephropathy = Yes ⟹ DFU	0.02	1.00	2.33
11	Diabetic treatment = Injection, HDL = High, Systolic BP = hypertension ⟹ DFU	0.02	1.00	2.33
12	Addiction to other drugs = Yes, Physical activity = No, Systolic BP = Pre-hypertension, Non Proliferative retinopathy = Yes ⟹ DFU	0.02	1.00	2.33
13	Gender = Male, History of other diseases = Kidney & heart, HDL = High ⟹ DFU	0.02	1.00	2.33
14	Gender = Male, Age = 46–65 Years, Duration of diabetes = > 10 Years, Proliferative retinopathy = Yes ⟹ DFU	0.02	1.00	2.33
15	…			
74	Gender = Male, BMI = Obese, History of other diseases = Heart, Systolic BP = Pre-hypertension ⟹ DFU	0.01	1.00	2.33
75	BMI = Obese, Visiting the doctor regularly = No, LDL = Optimal, HDL = High ⟹ DFU	0.01	1.00	2.33

**Table 3:** Rule generated by the CBA algorithm for patients belongs to diabetes class.

#	Rules	Support	Confidence	Lift
1	Duration of diabetes = < 10 Years, Addiction to other drugs = No, LDL = Near optimal, Diabetic neuropathy = No ⟹ Diabetes	0.05	1.00	1.75
2	Duration of diabetes = < 10 Years, Diabetic treatment = Drug, LDL = Near optimal ⟹ Diabetes	0.04	1.00	1.75
3	Age = 26–45 Years, Diabetic treatment = Drug, Family history of diabetes = No, Systolic BP = Normal ⟹ Diabetes	0.03	1.00	1.75
4	Gender = Female, Duration of diabetes = < 10 Years, Family history of diabetes = No, LDL = Near optimal ⟹ Diabetes	0.03	1.00	1.75
5	LDL = Near optimal, TG = Normal, Nonproliferative retinopathy = No, Diabetic nephropathy = No ⟹ Diabetes	0.03	1.00	1.75
6	BMI = Under weight, Duration of diabetes = < 10 Years, Addiction to other drugs = No ⟹ Diabetes	0.02	1.00	1.75
7	BMI = Over weight, Duration of diabetes = < 10 Years, HDL = Low, Bs2hpp = Pre-diabetes ⟹ Diabetes	0.02	1.00	1.75
8	Gender = Female, BMI = Normal weight, Family history of diabetes = No, Cardiovascular events = Yes ⟹ Diabetes	0.02	1.00	1.75
9	Age = 26–45 Years, Duration of diabetes = < 10 Years, HDL = Low, Non proliferative retinopathy = No ⟹ Diabetes	0.02	1.00	1.75
10	Gender = Female, History of other diseases = No, Visiting the doctor regularly = No, HDL = Borderline high ⟹ Diabetes	0.02	1.00	1.75
11	Age = 26–45 Years, BMI = Normal weight, Diabetic treatment = Drug ⟹ Diabetes	0.02	1.00	
12	Gender = Female, HDL = Low, TC = Acceptable, TG = High ⟹ Diabetes	0.02	1.00	1.75
13	Gender = Female, History of other diseases = No, Family history of diabetes = No, Diastolic BP = Pre-hypertension ⟹ Diabetes	0.02	1.00	1.75
14	History of other diseases = No, Diastolic BP = Pre-hypertension, FBS = Diabetes, Non proliferative retinopathy = No ⟹ Diabetes	0.02	1.00	1.75
15	Diabetic treatment = Drug, Addiction to other drugs = No, LDL = Borderline high ⟹ Diabetes	0.02	1.00	1.75
. . .	. . .	. . .	. . .	. . .
70	Gender = Female, BMI = Normal weight, History of other diseases = Kidney, HDL = Low ⟹ Diabetes	0.01	1.00	1.75
71	Age = 26–45 Years, Diabetic treatment = Drug, HDL = Low, Diastolic BP = Hypertension ⟹ Diabetes	0.01	1.00	1.75

Checking the rules identified by the CBA algorithm in Table 2; duration of diabetes more than 10 years, insulin therapy, male sex, older age, being a smoker, addiction to other drugs, family history of diabetes, higher body mass index, physical inactivity, having proliferative, non-proliferative retinopathy, nephropathy, history of heart or kidney disease, level of LDL, HDL cholesterol, triglyceride (TG), systolic and diastolic blood pressure, BS2HPP and experience of cardiovascular events are effective in the occurrence of diabetic foot ulcer.

The overall accuracy of the CBA algorithm for identifying patients in two groups was equal to 96%. The accuracy for identifying patients with and without DFUs is 97% and 95%, respectively. Confusion matrix for a test data by CBA algorithm presented in Table 4. Also, the AUC value of the area under the ROC curve of the test data was 0.962 (95%CI 0.924, 1.000).

**Table 4:** Confusion matrix for the same test data by CBA algorithm and logistic regression model.

		CBA algorithm		Logistic regression	
		Actual class		Actual class	
		DFUs	No-DFUs	DFUs	No-DFUs
Predicted class	DFUs	60	2	49	17
	No-DFUs	3	69	14	54
Accuracy		96%		77.4%	
AUC		0.962 (95%CI 0.924, 1.000)		0.769 (95%CI 0.686, 0.852)	

*DFUs* diabetic patients with foot ulcers, *AUC* area under the ROC curve, *CBA* classification based on associations.

The overall accuracy and AUC of logistic regression for identifying patients in two groups is 77.4%. The accuracy for identifying patients with and without DFUs was 77.78% and 76%, respectively. The results showed that the CBA algorithm has a better performance in terms of accuracy and AUC than the logistic regression model in predicting DFUs.

Based on the results of the multiple regression model; duration of diabetes more than 10 years, insulin therapy, male sex, addiction to other drugs, family history of diabetes, higher body mass index, having non-proliferative retinopathy, nephropathy, history of heart or kidney disease, higher level of LDL cholesterol, HDL cholesterol, triglyceride (TG), significantly increase the chance of occurrence of diabetic foot ulcer.

**Figure 1:**
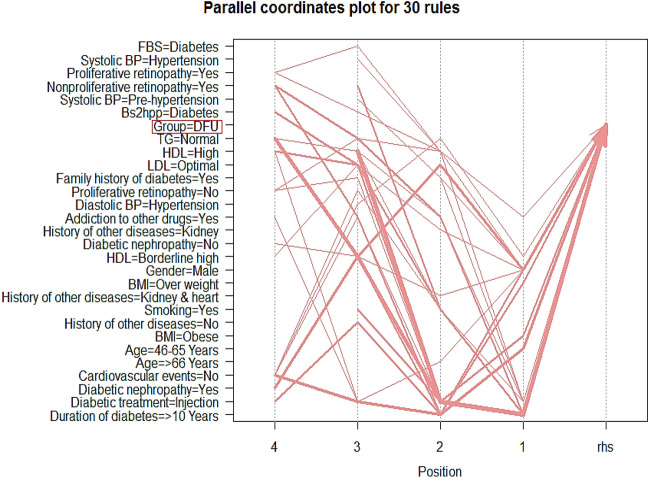
Parallel coordinate plot extracted based on CBA algorithm for DFUs.

**Figure 2:**
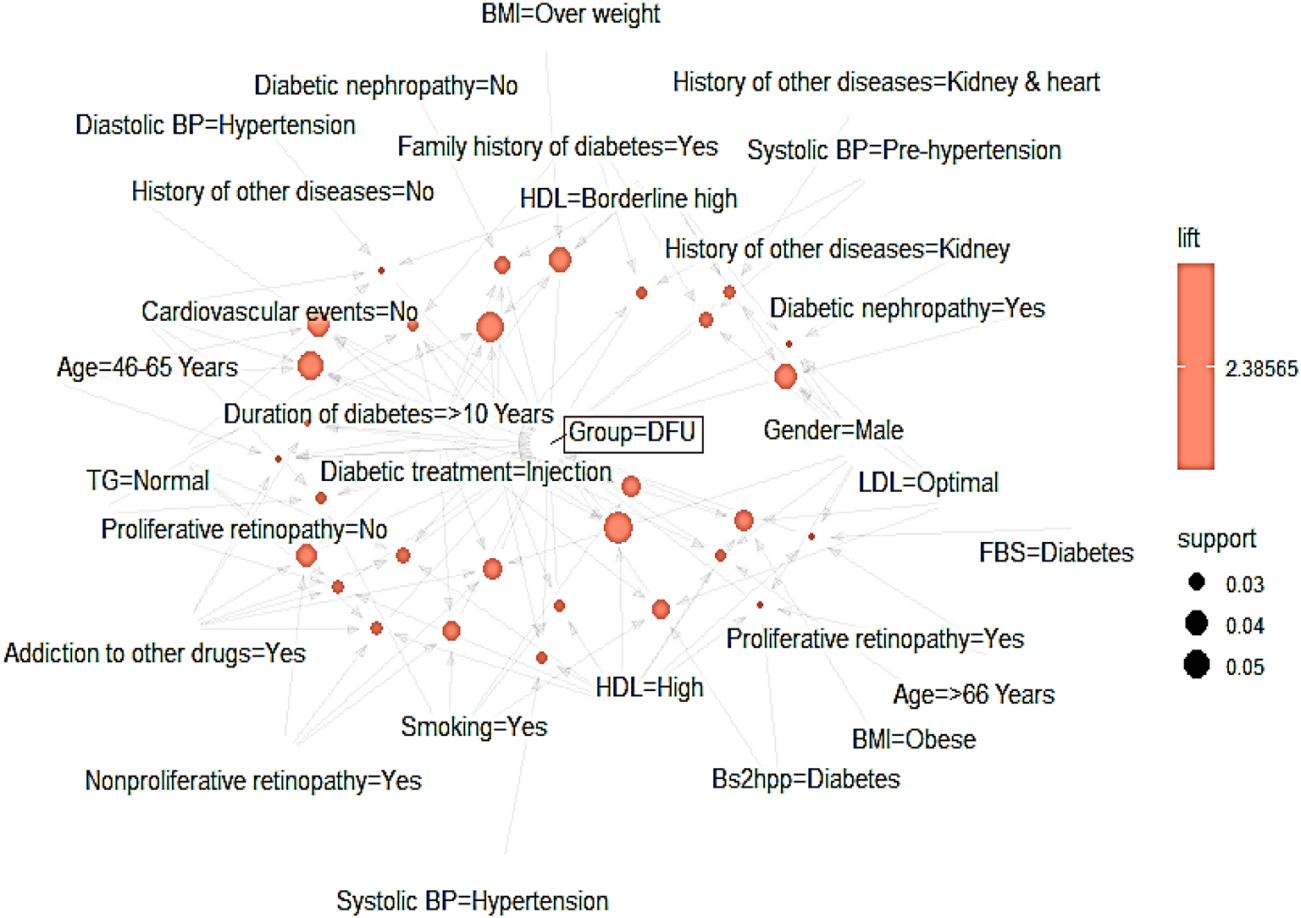
Graph-based visualization with items and rules as vertices extracted based on CBA algorithm for DFUs (this graph created using the “arulesViz” package based on the dataset of this study).

Graph-based visualization with items and rules as vertices extracted based on CBA method for DFUs was presented in Fig. 1. Also, parallel coordinate plot extracted based on CBA method for DFUs was reported in Fig. 2. In the given diagram, the red circles show the rules. The incoming arrows indicate the left-hand rules (lhs) and the outgoing arrows indicate the right-hand rules (rhs).

## Discussion

Foot ulceration is a widespread issue that comes with substantial healthcare expenses. As a severe complication of diabetes, DFUs have a significant impact on the well-being of patients. The study aimed to identify individuals with diabetes at risk of developing DFU by developing an associative classification-based model using the Apriori algorithm. The algorithm identifies several risk factors related to developing DFUs as long-term diabetes, insulin therapy, male gender, advanced age, smoking, drug addiction, family history of diabetes, higher BMI, physical inactivity, and diabetic complications. The CBA algorithm demonstrated high accuracy of 96% in identifying patients with and without DFUs. The CBA algorithm performs better in terms of accuracy and AUC than the logistic regression model in predicting DFUs. The variables identified by the two methods are very similar. All the variables that are significant in logistic regression are also identified as important variables in the rule generated based on the CBA model.

The study extracted interesting patterns from a real dataset using data mining, which is particularly useful in medical data due to the high volume of data and unknown relationships between factors. The patterns and models obtained can be used to generate hypotheses for subsequent studies, including clinical trials to confirm or refute them, ultimately improving evidence-based clinical studies.

Several studies have explored predictive models for DFUs, including a study by Jiang et al.^34^ They developed a nomogram that utilizes 12 easily obtainable risk factors, to predict the likelihood of DFUs in hospitalized patients with type 2 diabetes. The nomogram achieved an accuracy rate of 84% in predicting DFUs in validation cohorts. Identified risk factors for DFU include male gender, old age, longer duration of diabetes, history of foot disease, and various blood markers such as high white blood cell count and low hemoglobin level.

Shi et al.^35^ constructed potent weighted risk model using Random Forest algorithm for evaluating the occurrence DFUs. RF model based on 17 variables achieved the accuracy of o.795 for predicting risk of DFUs in external validation data sets.

Monteiro-Soare et al.^36^ developed a risk stratification model for DFUs using seven commonly available clinical variables such as age, gender, duration of diabetes, HbA1c levels, neuropathy, peripheral arterial disease, and previous history of foot ulcers. In this particular study, 336 patients with diabetes were enrolled and monitored for a median duration of 2.3 years to investigate the incidence of new DFU as the primary outcome. The study found that a logistic regression model achieved an area under the curve (AUC) of 0.83.

Lv et al.^37^ developed a nomogram that utilized a logistic regression model to predict the risk of DFUs. According to their findings, risk factors for foot ulcers included abnormal foot skin color, callus, BMI, foot arterial pulse, and a history of ulcers. The validation of the nomogram demonstrated moderate predictive ability, as shown by an AUC value of 0.787.

Research on DFUs has often focused on identifying risk factors for their development in diabetic patients. The present study supports previous research indicating a link between male gender and the occurrence of DFUs. Several other investigations, including those by Larijani et al., Bejestani et al., Ali et al., Jiang et al., Bakri et al., Frikberg et al., Richard et al., and Finke et al., have also found a higher proportion of men developing DFUs. This may be due to the higher pressure on men’s lower limbs due to their average weight, as well as differences in lifestyle and self-care^34,38–44^. The higher prevalence of atherosclerosis in men compared to women, as noted in a study by JanMohammadi et al.^45^ may also contribute to the higher rate of DFUs in men.

The mean age of patients with DFUs was considerably higher than that of patients without DFUs. This result is consistent with the findings reported by Shahi et al., Zhang et al., Yunir et al., and Jiang et al., who identified an age over 50 years as a significant risk factor for the development of foot ulcers in diabetic patients^34,46–48^.

The present study established a significant correlation between the longer duration of diabetes and the risk of developing DFU. Specifically, 56% of patients with DFUs had a history of diabetes for more than 10 years, while only 28% of diabetic patients without foot ulcers had a history of diabetes for more than 10 years. These results are consistent with the findings of several other investigations, such as those conducted by Bakri et al., Syauta et al., Frikberg et al., Lipsky et al., and Naemi et al. In Bejestani et al.’s study, 50% of patients had a history of diabetes for over 13 years, in Ali et al.’s study, 58% for over 10 years, and in Chowdhury et al.'s study, 56% for over 10 years^39–42,49–52^.

The present study also found a statistically significant relationship between physical activity and the incidence of DFUs, which was confirmed in Tola et al.’s study^53^.

A history of smoking or other drug addiction was associated with the development of DFUs. This finding is consistent with the results of other studies, including those by Bejestani et al., Syauta et al., Frikberg et al., Naemi et al., and Moeini et al., which identified smoking and other drugs as risk factors for DFU^39,42,49,51,54^.

In the studies conducted by Reardon et al.^55^ and Abu Obaid et al.^56^ regarding the factors affecting DFUs, a statistically significant relationship was found between the occurrence of foot ulcers and regular visits to the doctor. These results are in agreement with the findings of the present study.

The results of this study indicate a positive correlation between increased BMI and the occurrence of DFUs. This finding is consistent with previous research that has identified obesity and elevated BMI as potential risk factors for diabetic foot ulcers. In fact, in our study, we found that 87.1% of the patients were either overweight or obese, while only 12.9% had a normal weight. None of the patients in our study were underweight. It is generally observed that diabetic patients with elevated BMI have a higher incidence of DFUs.

To the best of the present study's knowledge, there has been no research conducted on the application of association rules mining specifically in patients with DFUs. However, several studies have used association rule mining techniques to explore the relationship between various risk factors and diabetes. Rane and Rano^57^, for example, employed an association rule exploration algorithm on recorded information of diabetic patients to determine the frequent risk factors associated with the occurrence of diabetes. Their findings indicated that in most of the obtained rules, low levels of HDL cholesterol (less than 35 mg/dL) were the most likely factor associated with the occurrence of diabetes.

In a study conducted in 2010 by Patil et al.^58^ a hidden pattern discovery algorithm was implemented on various variables of 625 female diabetic patients. The results confirmed that blood sugar levels greater than 150 mg/deciliter, age between 40 and 60 years, body mass index greater than 30 kg/m^2^, and pregnancy frequency greater than 5 times had the greatest association with the occurrence of diabetes.

This study developed predictive models utilizing readily available routine features. As a result, primary clinics can utilize the constructed CBA predictive model to screen patients with diabetes mellitus for their susceptibility to developing DFUs, even in cases where physicians lack experience. However, due to the retrospective nature of the study design and the data extraction procedure, some of the laboratory data were missing or were not available. Therefore, future work should analyses the potential benefit of adding other variables to those routinely recorded. This study also does not allow establishing the temporal sequence between selected risk factors and the occurrence of DFUs. Future prospective studies are needed to establish this association.

### Study limitation

While the method achieved a higher overall accuracy compared to other studies, it is crucial to validate and replicate the results in other databases to ensure their generalizability to diverse populations. Future research on this topic should prioritize larger sample sizes and multi-center studies to enhance our understanding of the risk factors associated with DFUs and improve the accuracy of predictive models.

## Data Availability

The datasets used and/or analysed during the current study available from the corresponding author on reasonable request.

## References

[1] Zhang P (2017). Global epidemiology of diabetic foot ulceration: A systematic review and meta-analysis. Ann. Med..

[2] Raghav A (2018). Financial burden of DFUsto world: A progressive topic to discuss always. Ther. Adv. Endocrinol. Metab..

[3] Deshpande AD, Harris-Hayes M, Schootman M (2008). Epidemiology of diabetes and diabetes-related complications. Phys. Ther..

[4] Hicks CW (2020). Incidence and risk factors associated with ulcer recurrence among patients with DFUstreated in a multidisciplinary setting. J. Surg. Res..

[5] Shen JF (2020). Recurrence and influencing factors of diabetic foot ulcer in patients with type 2 diabetes mellitus. Zhonghua Shao Shang ZaZhi.

[6] McDermott K, Fang M, Boulton AJM, Selvin E, Hicks CW (2023). Etiology, epidemiology, and disparities in the burden of diabetic foot ulcers. Diabetes Care.

[7] Chen L, Sun S, Gao Y, Ran X (2023). Global mortality of diabetic foot ulcer: A systematic review and meta-analysis of observational studies. Diabetes Obes. Metab..

[8] Martins-Mendes D (2014). The independent contribution of diabetic foot ulcer on lower extremity amputation and mortality risk. J. Diabetes Complicat..

[9] Armstrong DG, Boulton AJM, Bus SA (2017). DFUs and their recurrence. N. Engl. J. Med..

[10] Prompers L (2007). High prevalence of ischaemia, infection and serious comorbidity in patients with diabetic foot disease in Europe. Baseline results from the Eurodiale study. Diabetologia.

[11] Bender C (2022). Using case-based reasoning in a learning system: A prototype of a pedagogical nurse tool for evidence-based diabetic foot ulcer care. J. Diabetes Sci. Technol..

[12] Chen D (2021). Development and validation of an incidence risk prediction model for early foot ulcer in diabetes based on a high evidence systematic review and meta-analysis. Diabetes Res. Clin. Pract..

[13] Zhou Q (2018). Development and validation of a brief diabetic foot ulceration risk checklist among diabetic patients: A multicenter longitudinal study in China. Sci. Rep..

[14] Tomita M (2015). Development and assessment of a simple scoring system for the risk of developing diabetic foot. Diabetol. Int..

[15] Wu SC, Driver VR, Wrobel JS, Armstrong DG (2007). Foot ulcers in the diabetic patient, prevention and treatment. Vasc. Health Risk Manag..

[16] Al-Mohaithef M, Abdelmohsen SA, Algameel M, Abdelwahed AY (2022). Screening for identification of patients at high risk for diabetes-related foot ulcers: A cross-sectional study. J. Int. Med. Res..

[17] Boyko EJ, Ahroni JH, Cohen V, Nelson KM, Heagerty PJ (2006). Prediction of diabetic foot ulcer occurrence using commonly available clinical information: The Seattle diabetic foot study. Diabetes Care.

[18] Monteiro-Soares M, Boyko EJ, Ribeiro J, Ribeiro I, Dinis-Ribeiro M (2011). Risk stratification systems for diabetic foot ulcers: A systematic review. Diabetologia.

[19] Ehrmann DE, Joshi S, Goodfellow SD, Mazwi ML, Eytan D (2023). Making machine learning matter to clinicians: Model actionability in medical decision-making. NPJ Digit. Med..

[20] Sutton RT (2020). An overview of clinical decision support systems: Benefits, risks, and strategies for success. NPJ Digit. Med..

[21] Soni J, Ansari U, Sharma D, Soni S (2011). Predictive data mining for medical diagnosis: An overview of heart disease prediction. Int. J. Comput. Appl..

[22] Pazhanikumar K, Arumugaperumal S (2013). Association rule mining and medical application: A detailed survey. Int. J. Comput. Appl..

[23] Nahar J, Imam T, Tickle KS, Chen YPP (2013). Association rule mining to detect factors which contribute to heart disease in males and females. Expert Syst. Appl..

[24] Zhang W, Ma D, Yao W (2014). Medical diagnosis data mining based on improved Apriori algorithm. J. Netw..

[25] ElSayed NA (2023). American diabetes association. 2. Classification and diagnosis of diabetes: Standards of care in diabetes—2023. Diabetes Care.

[26] Bus SA (2020). International working group on the diabetic foot. Guidelines on the prevention of foot ulcers in persons with diabetes (IWGDF 2019 update). Diabetes Metab. Res. Rev..

[27] Lavery LA (2016). WHO guidelines update: Diabetic foot ulcer treatment guidelines. Wound Repair Regen..

[28] Pitocco D (2019). Diabetic foot infections: A comprehensive overview. Eur. Rev. Med. Pharmacol. Sci..

[29] Wagner FW (1979). A classification and treatment program for diabetic, neuropathic, and dysvascular foot problems. Instr. Course Lect..

[30] Aggarwal, C. C. An introduction to frequent pattern mining. In *Frequent Pattern Mining* 1–17 (Springer, 2014).

[31] Borgelt, C. & Kruse, R. Induction of association rules: Apriori implementation. In *Compstat: Proceedings in Computational Statistics* 395–400 (Physica-Verlag HD, 2002).

[32] Liu, B., Ma, Y. & Wong, C. K. Classification using association rules: Weaknesses and enhancements. In *Data Mining for Scientific and Engineering Applications* 591–605 (Springer, 2001).

[33] Chen G, Liu H, Yu L, Wei Q, Zhang X (2006). A new approach to classification based on association rule mining. Decis. Support Syst..

[34] Jiang M (2022). Predicting the risk of DFUs from diabetics with dysmetabolism: A retrospective clinical trial. Front. Endocrinol..

[35] Shi L (2021). A potent weighted risk model for evaluating the occurrence and severity of diabetic foot ulcers. Diabetol. Metab. Syndr..

[36] Monteiro-Soares M, Dinis-Ribeiro M (2010). External validation and optimisation of a model for predicting foot ulcers in patients with diabetes. Diabetologia.

[37] Lv J (2023). Development and validation of a risk prediction model for foot ulcers in diabetic patients. J. Diabetes Res..

[38] Larijani B (2001). Lower limb amputation rate in patients with type 2 diabetes managed at the Imam Khomeiny and Doctor Shariati hospitals between 1979 and 1994. Iran. J. Diabetes Metab..

[39] Shahrad BH, Motabar A (2004). Assessment of diabetic foot ulcer’s predisposing factors and its outcomes in patients with diabetic foot syndrome hospitalized in HazratRasoul-e-Akram Hospital in Tehran during 1996–2001. Razi J. Med. Sci..

[40] Ali SM, Basit A, Sheikh T, Mumtaz S, Hydrie M (2001). Diabetic foot ulcer: A prospective study. JPMA J. Pak. Med. Assoc..

[41] Bakri FG, Allan AH, Khader YS, Younes NA, Ajlouni KM (2012). Prevalence of diabetic foot ulcer and its associated risk factors among diabetic patients in Jordan. J. Med. J..

[42] Frykberg RG (1998). Role of neuropathy and high foot pressures in diabetic foot ulceration. Diabetes care.

[43] Richard JL (2011). Management of patients hospitalized for diabetic foot infection: Results of the French OPIDIA study. Diabetes Metab..

[44] Fincke BG, Miller DR, Turpin R (2010). A classification of diabetic foot infections using ICD-9-CM codes: Application to a large computerized medical database. BMC Health Serv. Res..

[45] Janmohammadi N, Moazzezi Z, Ghobadi P, Haddadi R, Montazeri M (2009). Evaluation of the risk factors of diabetic foot ulcer and its treatment in diabetic patients, Babol, north of Iran. Iran. J. Endocrinol. Metab..

[46] Shahi SK (2012). Prevalence of diabetic foot ulcer and associated risk factors in diabetic patients from North India. J. Diabet. Foot Complicat..

[47] Zhang L, Liu M (2022). Analysis of diabetes disease risk prediction and diabetes medication pattern based on data mining. Comput. Math. Methods Med..

[48] Yunir E, Hidayah CD, Harimurti K, Kshanti IAM (2022). Three years survival and factor predicting amputation or mortality in patients with high risk for diabetic foot ulcer in Fatmawati General Hospital, Jakarta. J. Prim. Care Community Health.

[49] Syauta D, Hendarto J, Mariana N, Kusumanegara J, Faruk M (2021). Risk factors affecting the degree of DFU saccording to Wagner classification in diabetic foot patients. MedicinaClínicaPráctica.

[50] Lipsky BA (2020). Guidelines on the diagnosis and treatment of foot infection in persons with diabetes (IWGDF 2019 update). Diabetes/Metab. Res. Rev..

[51] Naemi R, Chockalingam N, Lutale JK, Abbas ZG (2020). Predicting the risk of futurediabetic foot ulcer occurrence: A prospective cohort study of patients with diabetes in Tanzania. BMJ Open Diabetes Res. Care.

[52] Chowdhury HK, Khan MH, Wadud JR (2000). Risk factors for the development of diabetic foot ulcer in Bangladesh. Diabetes Res. Clin. Pract..

[53] Tola A, Regassa LD, Ayele Y (2021). Prevalence and associated factors of DFUsamong type 2 diabetic patients attending chronic follow-up clinics at governmental hospitals of Harari Region, Eastern Ethiopia: A 5-year (2013–2017) retrospective study. SAGE Open Med..

[54] Moeini M, Shahriari M, Yousefi H, Esfandiari G, Babaahmadi M (2017). An investigation on the wound severity and its association with predisposing factors in patients with diabetic foot. J. Clin. Nurs. Midwifery.

[55] Reardon R (2020). The diabetic foot ulcer. Aust. J. Gen. Pract..

[56] Obaid HAA, Eljedi A (2015). Risk factors for the development of DFUs in Gaza Strip: A case-control study. Age (Omaha).

[57] Rane N, Rao M (2013). Association rule mining on type 2 diabetes using FP-growth association rule. Int. J. Eng. Comput. Sci..

[58] Patil, B. M., Joshi, R. C. & Toshniwal, D. Association rule for classification of type-2 diabetic patients. In *2010 Second International Conference on Machine Learning and Computing* 330–334 (2010).

